# Impact of COVID-19 on hospital screening, diagnosis and treatment activities among prostate and colorectal cancer patients in Canada

**DOI:** 10.1007/s10754-023-09342-3

**Published:** 2023-04-02

**Authors:** Shin-Haw Lee, Andrew Toye Ojo, Matthew Halat, Nataly Bleibdrey, Steven Zhang, Rob Chalmers, Dan Zimskind

**Affiliations:** ZS Associates, Toronto, Canada

**Keywords:** COVID-19, Colorectal Cancer, Prostate Cancer, Cancer Screening, Cancer, Treatment, I18

## Abstract

**Background:**

Suspension of cancer screening and treatment programs were instituted to preserve medical resources and protect vulnerable populations. This research aims to investigate the implications of COVID-19 on cancer management and clinical outcomes for patients with prostate and colorectal cancer in Canada.

**Methods:**

We examined hospital cancer screening, diagnosis, treatment, length of stay, and mortality data among prostate and colorectal cancer patients between April 2017 and March 2021. Baseline trends were established with data between April 2017 and March 2020 for comparison with data collected between April 2020 and March 2021. Scenario analyses were performed to assess the incremental capacity requirements needed to restore hospital cancer care capacities to the pre-pandemic levels.

**Results:**

For prostate cancer, A 12% decrease in diagnoses and 5.3% decrease in treatment activities were observed during COVID-19 between April 2020 and March 2021. Similarly, a 43% reduction in colonoscopies, 11% decrease in diagnoses and 10% decrease in treatment activities were observed for colorectal cancers. An estimated 1,438 prostate and 2,494 colorectal cancer cases were undiagnosed, resulting in a total of 620 and 1,487 unperformed treatment activities for prostate and colorectal cancers, respectively, across nine provinces in Canada. To clear the backlogs of unperformed treatment procedures will require an estimated 3%-6% monthly capacity increase over the next 6 months.

Interpretation.

A concerted effort from all stakeholders is required to immediately ameliorate the backlogs of cancer detection and treatment activities. Mitigation measures should be implemented to minimize future interruptions to cancer care in Canada.

**Supplementary Information:**

The online version contains supplementary material available at 10.1007/s10754-023-09342-3.

## Introduction

Because early detection of cancer through routine screening is a crucial driver of improved cancer survival rates, it is imperative that all eligible patients receive timely screening, which is not currently happening systematically across Canada (Bryan et al., 2018; Diaz-Tasende, 2018; Emery et al., 2013). Minimizing delayed cancer diagnosis has proven critical as timely removal of neoplastic lesions significantly reduces treatment complexities for cancer patients and lessens the burden on primary care providers. While recent advances in cancer screening technologies such as omics- and serum-based testing for prostate cancer and fecal immunochemical testing for colorectal cancer have made cancer screening more accessible, not all screen-eligible individuals are being screened every year (Clarke & Feuerstein, 2019; Ladabaum et al., 2020; Tan et al., 2019; Tsujino et al., 2021). Therefore, understanding the full range of performance from screening to diagnosis to treatment and ultimately mortality will be valuable to inform future decision-making in improving cancer management and care.

Disruptions to healthcare access may negatively impact early detection of cancers and lead to a surge of advanced cancer cases that could overwhelm the healthcare system and lead to poorer treatment outcomes (Sung et al., 2021). A recent study showed that a 4-week delay in cancer treatment is associated with a 10% increase in mortality across surgical, systemic, and radiotherapy treatments (Hanna et al., 2020a). In Canada, patients with prostate cancer have reported prolonged wait-times between diagnosis and treatment (Tran et al., 2015). Similarly, an average wait-time of 86 days to endoscopy has been reported for colorectal cancer patients, considerably longer than the recommended guideline of 60 days (Janssen et al., 2016). Nevertheless, reducing cancer diagnostic exam wait-times has been a recent focus of cancer care in Canada.

In March 2020, a nationwide postponement of non-urgent medical procedures and services was instituted to preserve healthcare resources for the coronavirus disease 2019 (COVID-19) pandemic. Mounting evidence has demonstrated significant decreases in the number of cancer screenings and diagnoses during the pandemic (Dee et al., 2020; Fonseca et al., 2021; Kaufman et al., 2020). This observation of delays in cancer screening and therefore diagnosis could lead to a devastating surge of advanced cancer cases that would seriously compromise our healthcare systems. Similar restrictions have impacted the spectrum of cancer care globally (Hanna et al., 2020b; Maringe et al., 2020; Patt et al., 2020; Richards et al., 2020). Therefore, a comprehensive assessment of the Canadian prostate cancer and colorectal cancer landscapes is required to fully understand the impact of COVID-19 on the Canadian oncology environment, specifically for prostate and colorectal cancers.

## Methods

### Data source

We employed datasets from the Canadian Institute for Health Information (CIHI) (https://www.cihi.ca/en) on prostate and colorectal cancer screening, diagnoses, treatment, and mortality events quarterly from April 2010 to March 2021, with breakdown by age group (< 40, 40–59, 60–79, 80 +) and province (with the exception of British Columbia and Quebec due to opt-outs). Specifically, CIHI’s Discharge Abstract Database (https://www.cihi.ca/en/discharge-abstract-database-metadata-dad) and National Ambulatory Care Reporting System (https://www.cihi.ca/en/national-ambulatory-care-reporting-system-metadata-nacrs) metadata captures ICA-10-CA- and CCI-based administrative, day surgery, and clinical information on hospital discharges directly from acute care facilities or their respective health authorities. In the current study, diagnosis and treatment data included data breakdown by stage of cancer (non-metastatic vs metastatic). Moreover, treatment data included overall data on treatment classes (imaging, surgery, radiotherapy, pharmacotherapy) along with specific treatment interventions performed. Additionally, key COVID-19 intervention data by federal, provincial and territorial governments (i.e. closures of non-essential businesses, postponement of non-essential health services, state of emergency announcements, and vaccine implementation milestones) were obtained from CIHI’s publicly available COVID-19 Intervention Timeline in Canada dataset (https://www.cihi.ca/en/covid-19-intervention-timeline-in-canada). The current study was approved by the CIHI’s Decision Support Services and ZS Associates Research & Development Excellence Committee.

### Study design and statistical rationale

A retrospective cohort study involving nine provinces in Canada was conducted. Specifically, aggregate, ICD code-based, semi-annual prostate and colorectal cancer patient journey datasets from 2010 to 2020 with breakdown by age group, disease stage, and province were obtained from CIHI whereby addition of all independent aggregate data (screening events, hospital admissions, treatment activities, patient expirations) or averaging of all independent aggregate data (median length of stay) was performed in half-year intervals. To understand the baseline screening, diagnosis, treatment activities, length of stay, and mortality among prostate and colorectal cancer patients prior to the COVID-19 pandemic, analysis was first carried out using data from April 2010 to March 2020. Subsequent inclusion of data from April 2020 to March 2021 in our analysis allowed the direct comparison of reported data pre- and during COVID-19. We also calculated 5-year averages from April 2010 to March 2015 and April 2015 to March 2020 as a baseline for pre-COVID-19 trend assessment and statistical analyses for each outcome. All descriptive statistical analyses were performed using R, version 4.2.0 (The R Foundation for Statistical Computing, Vienna) and data are reported and shown as mean ± SEM unless otherwise indicated. Specifically, two-tailed t tests were used to analyze normally distributed data for each mean comparison in our study. Furthermore, normality of the data distribution was tested using the Shapiro–Wilk’s test. A dataset with a *p* value > 0.05 from the Shapiro–Wilk’s normality test was considered to follow a normal distribution. In situations where the data is not normally distributed (a *p* value < 0.05 from the Shapiro–Wilk’s normality test), Mann–Whitney U test, a non-parametric test, was used for the mean comparison. Statistical comparison was considered significant at the *p* ≤ 0.05 significance level.

### Data availability

Available aggregate hospital data for prostate and colorectal cancer screening, diagnosis, treatment activities, hospital length of stay, and mortality were obtained using the most responsible ICD codes from CIHI. Detailed specification of each ICA-10-CA/CCI code used in the current is presented in Supplemental Table 9. The median length of stay was calculated where at least one of the ICD-10-CA most responsible diagnosis codes listed above was identified for prostate and colorectal cancer. The number of patient expirations was reported where a most responsible diagnosis code listed above for prostate and colorectal cancer was identified at the time of expiration. The analysis did not include data where age was unknown nor data from out of hospital (private/clinic practices) and abandoned procedures. Lasty, to comply with CIHI’s data privacy and confidentiality policies, in instances in which there were fewer than five reported cases, the reported values were suppressed to avoid residual disclosure of the identity of individuals or health facilities.

## Results

### Temporal analysis of hospital screening and admission events uncovers delays in access to cancer care during COVID-19

**Fig. 1:**
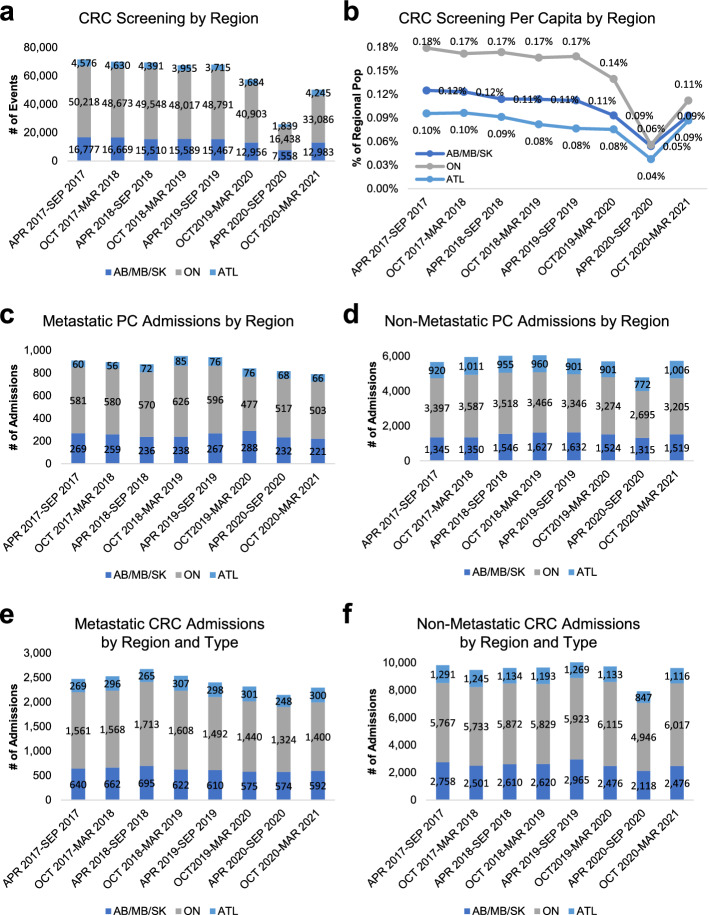
Temporal trends in hospital prostate and colorectal cancer screening and admissions from April 2017 to March 2021. **a**, **b** Temporal analysis of hospital colorectal screening events by region on an absolute value **a** and a per capita **b** basis. **c**, **d** Temporal analysis of prostate cancer hospital admissions showing the total number of hospital admissions for metastatic **c** and non-metastatic **d** prostate cancer by region. **e**, **f** Temporal analysis of colorectal cancer hospital admissions showing the total number of hospital admissions for metastatic **e** and non-metastatic **f** colorectal cancer by region. Population estimates for the indicated regions/provinces were extracted from Statistics Canada (https://www.statcan.gc.ca/en/subjects-start/population_and_demography) for the specified periods

To establish the baseline trends in hospital cancer screening, admission, and treatment activities prior to the COVID-19 pandemic, we utilized data from April 2017 to March 2020 to assess the general trends and variations across the Canadian prostate and colorectal cancer landscapes. A 6-month average of 67,345 ± 2,047 colorectal hospital screening events and 6,779 ± 75 prostate and 12,226 ± 82 colorectal cancer hospital admissions were reported from April 2017—March 2020 prior to COVID-19 (Supplemental Table 1–3). To determine the up-to-date trends in cancer screening, we have excluded hospital prostate cancer screening data from our analysis due to the wide practice of performing the prostate specific antigen (PSA) blood test outside of the hospital setting. Nevertheless, the summarized data is presented in Supplemental Table 1. With the nationwide suspension of non-urgent medical procedures and services in March 2020 to increase COVID-19 patient intake capacity, a marked decrease in colorectal screening events (*p* < 0.0001 compared to baseline) as well as prostate (*p* < 0.0001 compared to baseline) and colorectal cancer hospital admissions (*p* < 0.0001 compared to baseline) was observed between April 2020 and September 2020 (Fig. 1a, e–f). At the regional level, a significant reduction in colorectal cancer screening was observed in AB/MB/SK (*p* = 0.006 compared to baseline) where an outbreak of COVID-19 was recorded between October 2020 and March 2021 (Supplemental Table 1). Notably, regional differences in colorectal cancer screening coverage on a per capita basis were observed with AB/MB/SK and the Atlantic provinces trailing behind ON by 0.05%-0.08% in population coverage (Fig. 1b).

Our analysis also indicated that a greater impact was reported for non-metastatic hospital admissions among prostate (9% decrease in metastatic vs 19% decrease in non-metastatic hospital admissions) and colorectal (13% decrease in metastatic vs 19% decrease in non-metastatic hospital admissions) cancer patients across all regions during the initial 6 months of COVID-19 (Supplemental Table 2, 3 & Fig. 1d, f). For prostate cancer, a significant decrease in metastatic hospital admissions was observed throughout in AB/MB/SK and ON (Supplemental Table 2). For colorectal cancers, a marked decrease in both metastatic and non-metastatic hospital admissions was observed across all regions between April 2020 and September 2020, and a quick recovery in hospital admissions was reported between October 2020 and March 2021 with non-metastatic hospital admission in ON and metastatic hospital admission in ATL exceeding its baselines (Supplemental Table 3). Interestingly, an unexpected increase in the number of non-metastatic colorectal cancer cases above baseline in the < 40 age group was observed during COVID-19 despite significantly reduced screening effort during this time.

**Fig. 2:**
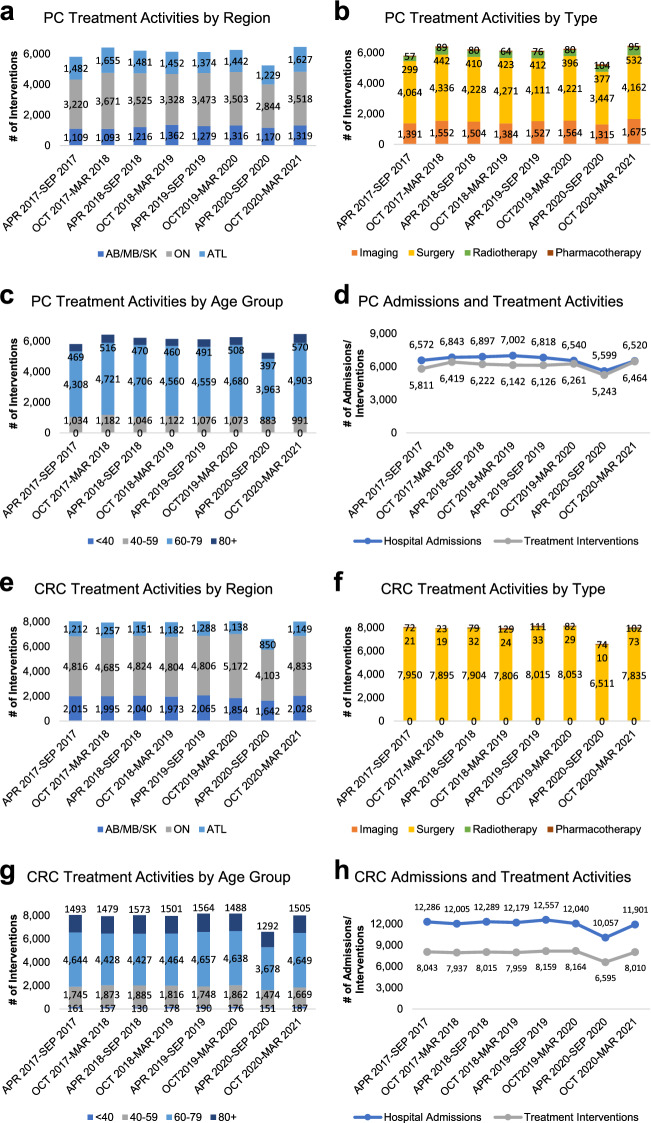
Temporal trends in hospital prostate and colorectal cancer treatment activities from April 2017 to March 2021. **a**–**c** Temporal analysis of prostate cancer hospital interventions demonstrating reported numbers interventional procedures by region **a**, intervention type **b**, and age group **c**. **d** Line chart depicting the relationship between prostate cancer hospital admissions and hospital treatment interventions from April 2017 to March 2021. **e**–**g** Temporal analysis of colorectal cancer hospital interventions demonstrating reported numbers interventional procedures by region **a**, intervention type **b**, and age group **c**. **h** Line chart depicting the relationship between colorectal cancer hospital admissions and hospital treatment interventions from April 2017 to March 2021

We next performed a detailed landscape analysis to assess hospital treatment activities for prostate and colorectal cancers (Supplemental Table 4, 5). Between April 2017 and March 2020, a 6-month average of 1,487 ± 33 (24%) imaging-, 4,205 ± 41 (68%) surgery-, 397 ± 21 (6%) radiotherapy-, and 74 ± 5 (1%) pharmacotherapy-based interventions were reported for prostate cancer patients (Supplemental Table 4). Similarly, a 6-month average of 7,937 ± 36 (98%) surgery-, 26 ± 2 (0.003%) radiotherapy-, and 83 ± 15 (0.01%) pharmacotherapy-based interventions were reported for colorectal cancer patients (Supplemental Table 5). For prostate cancer, a significant decrease in imaging- and surgery-based interventions was observed in ON and ATL while AB/MB/SK managed to minimize the impact of COVID-19 on surgical and imaging treatment activities (Fig. 2a–c). Interestingly, a significant increase in radiotherapy and pharmacotherapy above its baselines was reported in ON, suggesting potential changes in patient treatment plans during COVID-19. In line with this observation, a narrowing gap between hospital admissions and treatment activities was observed for prostate cancer during COVID-19 (Fig. 2d). For colorectal cancers, a significant decrease in surgical interventions was observed across all regions during the initial months of COVID-19, nonetheless, a ~ 99% of surgical intervention volume was restored between October 2020 and March 2021 (Fig. 2e–g and Supplemental Table 5). Of note, a gradual increase in radiotherapies and pharmacotherapies above its historical baselines for colorectal cancers was reported in ON during COVID-19. A consistent gap between colorectal cancer hospital admissions and treatment activities was observed from April 2017 to March 2021, suggesting the possibility of treatment activities performed outside of the hospital setting (Fig. 2h).

### Implications on ongoing care: early discharge of cancer patients and decreased hospital cancer mortality during the pandemic

**Fig. 3:**
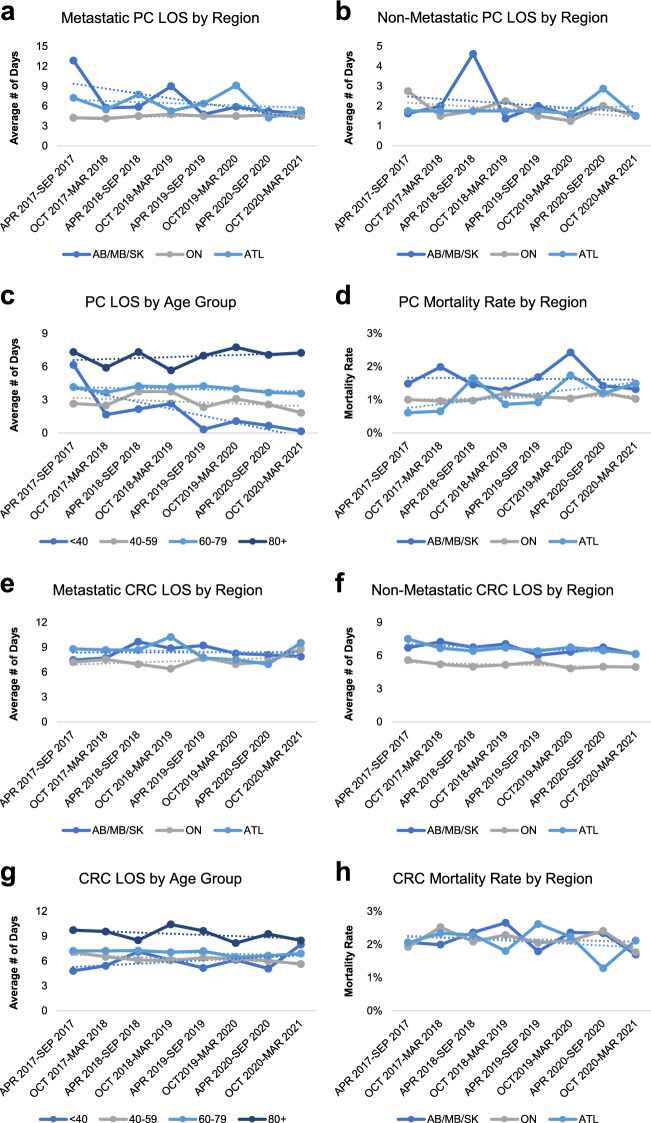
Temporal trends in prostate and colorectal cancer median calculated hospital length of stay and mortality rate from April 2017 to March 2021. **a**–**c** Temporal analysis of prostate cancer median calculated hospital length of stay demonstrating the average median calculated length of stays for metastatic **a** and non-metastatic prostate cancer **b** as well as trends by age group **c**. **d** Temporal analysis of hospital prostate cancer mortality rate demonstrating the reported numbers of hospital expirations by region from April 2017 to March 2021. **d**–**f** Temporal analysis of colorectal cancer median calculated hospital length of stay demonstrating the average median calculated length of stays for metastatic **a** and non-metastatic colorectal cancer **b** as well as trends by age group **c**. **d** Temporal analysis of hospital colorectal cancer mortality rate demonstrating the reported numbers of hospital expirations by region from April 2017 to March 2021. Mortality rate was calculated by dividing the total reported number of hospital expirations by the total number of hospital admissions for each specified period

Our analysis showed that there were natural variations in the median calculated hospital length of stay (LOS) for both prostate and colorectal cancers (Fig. 3a, b, e, f). Between April 2017 and March 2020, a 6-month average median calculated hospital LOS of 6.2 ± 0.4 days for metastatic and 1.9 ± 0.2 days for non-metastatic prostate cancer was reported (Supplemental Table 6). Similarly, a 6-month average of median calculated hospital LOS of 8.1 ± 0.1 days for metastatic and 6.2 ± 0.1 days for non-metastatic colorectal cancers was reported (Supplemental Table 7). While it is expected that metastatic cancer cases would require an extended hospital stay, our analysis showed that hospitals in ON consistently reported a lower hospital LOS than hospitals in AB/MB/SK and ATL for metastatic prostate cancer and non-metastatic colorectal cancers, albeit not significant (Fig. 3a, f).

To assess the variations in hospital LOS among prostate and colorectal cancer patients during COVID-19, we compared the median calculated LOS between April 2020 and March 2021 to its historical baseline data. For prostate cancer, a significant decrease of LOS was reported in ATL (Supplemental Table 6). An extended LOS was also reported for non-metastatic cancer patients likely from the 80 + age group in ATL between April 2020 and September 2020. For colorectal cancers, while a marked decrease in hospital LOS was observed for metastatic cancer patients in ATL, a significant increase in hospital LOS was observed for metastatic cancer patients in ON, suggesting a potentially overburdened cancer care system between October 2020 and March 2021 (Supplemental Table 7). Interestingly, our analysis also demonstrated that the hospital median calculated LOS increased proportionally as cancer patients aged from the 40–59 cohort to the 60–79 and 80 + cohorts (Fig. 3c, g). It is important to highlight that the changes in LOS observed in the current study are likely direct consequences of the precautionary COVID-19 measures and protocols instituted by the regional authorities. However, a follow-up investigation is warranted to examine whether LOS is impacted for cancer patients with delayed diagnoses.

Lastly, we assessed the changes in hospital patient mortality for prostate and colorectal cancers during COVID-19. Between April 2017 and March 2020, a 6-month average of 83 ± 4 prostate cancer patient expirations and 266 ± 6 colorectal cancer patient deaths were reported. For prostate cancer, a trend of decreasing patient hospital deaths was reported between April 2020 and March 2021. Similarly, a significant decrease in colorectal cancer patient hospital deaths across all regions was reported during COVID-19 with significantly fewer patient deaths reported from the 60–79 and 80 + age groups (Supplemental Table 8). Our mortality rate analysis showed that a trend of increasing prostate cancer mortality rate was observed in ATL over time whereas a relatively consistent, but slightly higher mortality was reported for colorectal cancers (Fig. 3d, h).

### Debt to pay: an estimated 2–16% monthly increase in hospital capacity is needed to clear unperformed cancer procedures

**Fig. 4:**
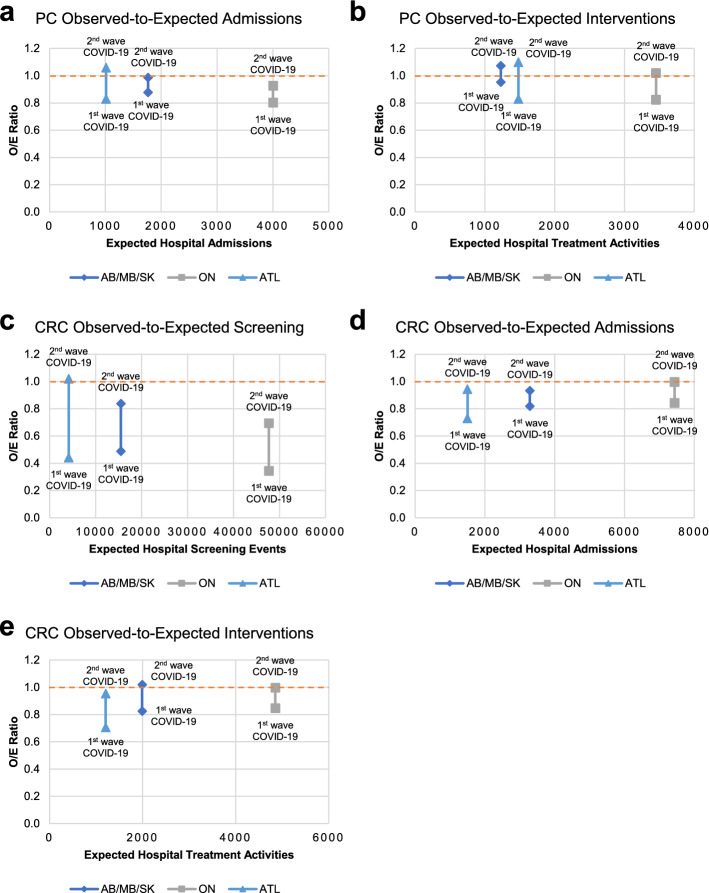
Observed-to-expected analysis of hospital prostate and colorectal cancer screening, admission, and treatment activities during the initial COVID-19 periods. **a**–**b** Scatter plots depicting prostate cancer observed-to-expected ratios of hospital admission **a**, and intervention **b** events by expected number of events (based on April 2017 to March 2020 data) for AB/MB/SK, ON, and ATL. **c**–**e** Scatter plots depicting colorectal cancer observed-to-expected ratios of hospital screening **c**, admission **d**, and intervention **e** events by expected number of events (based on April 2017 to March 2020 data) for AB/MB/SK, ON, and ATL. Orange dashed line represents when the number of observed events equals to the number of expected events (OE = 1)

To further investigate the impact of service interruption due to COVID-19, we next performed the observed-to-expected (OE) analysis for prostate and colorectal cancer hospital screening, admissions, and treatment activities during the first two waves of COVID-19 in Canada. Figure 4 demonstrated the total procedural deficits generated between April 2020 and March 2021. For prostate cancer hospital admissions, AB/MB/SK and ATL reported a total deficit of < 10% of their respective annual baselines while ON reported a total deficit of 14% of its annual baseline (Fig. 4a). For prostate cancer treatment activities, all regions reported a total deficit of < 5% of their annual baselines (Fig. 4b). Similarly, AB/MB/SK, ON, and ATL reported a total deficit of 34%, 49%, and 27%, respectively, for colorectal cancer screening (Fig. 4c), accumulating to an estimated total of 58,000 + unperformed colonoscopies in Canada. For colorectal cancer hospital admissions, our OE results indicated that a 13% deficit in AB/MB/SK, 8% deficit in ON, and 12% deficit in ATL were reported (Fig. 4d). In line with the reported admission deficits, our results showed that AB/MB/SK reported an 8% deficit in colorectal cancer treatment activities and ON and ATL reported an 8% and 12% deficit in colorectal cancer treatment activities, respectively (Fig. 4e). All in all, an increasing OE ratio close to 1, though a good sign for our cancer care system, is likely insufficient to restore a healthy and efficient treatment environment for cancer patients.

**Fig. 5:**
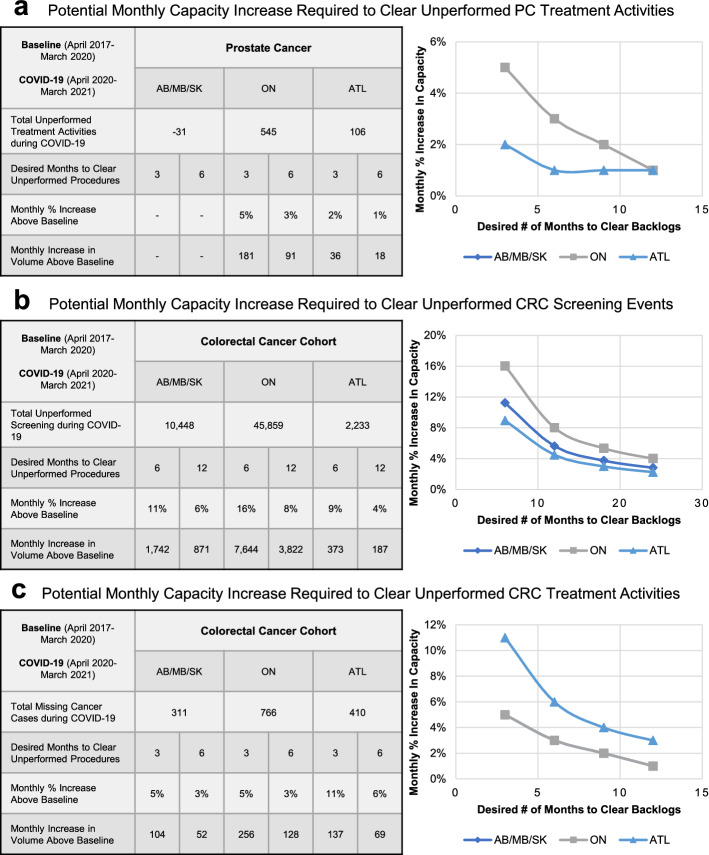
Estimation analysis of incremental hospital capacity for unperformed prostate and colorectal cancer procedures during the initial COVID-19 periods. **a** Table and line chart depicting the relationship between unperformed treatment activities for prostate cancer, potential monthly increase in hospital capacity above baseline, and time needed to clear unperformed procedures and treatment activities. **b**, **c** Table and line chart depicting the relationship between unperformed screening procedures **b** and treatment activities **c** for colorectal cancer, potential monthly increase in hospital capacity above baseline, and time needed to clear unperformed procedures and treatment activities. The calculations and estimations were performed using the formula: Monthly increase in volume = Total deficit during the COVID-19 period / Desired number of months to clear unperformed cases

More importantly, to help plan the incremental efforts required to take on all unperformed screening events and treatment procedures, an estimation analysis was performed to calculate the potential monthly capacity increase required to clear all unperformed cancer procedures (Fig. 5). Specifically, the potential monthly capacity increase as a percent of baseline was derived from dividing the total case deficit between April 2020 and March 2021 by the desired number of months to clear all unperformed procedures. Figure 5a demonstrated that ON and ATL reported a total of 545 and 106 unperformed treatment activities, resulting in a potential monthly capacity increase of 5% and 2% above baseline assuming a recovery time of 3-month is desired. Given the lower sense of urgency for clearing unperformed colonoscopies, an estimated monthly capacity increase of 11% in AB/MB/SK, 16% in ON, and 9% in ATL would be required to clear unperformed colonoscopies in 6 months (Fig. 5b). On the other hand, an estimated monthly hospital capacity increase of 5% in AB/MB/SK, 5% in ON, and 11% in ATL would be required to clear all unperformed colorectal cancer treatment activities in 3 months (Fig. 5c).

## Discussion

The COVID-19 pandemic has posed an unprecedented burden on our healthcare system and resulted in significant disruptions in cancer care in Canada. Accurately identifying and subsequently planning for the backlog of unperformed cancer screening and treatment activities during the pandemic is paramount to help restore timely diagnosis and treatment for cancer patients. In this study, we examined the impact of COVID-19 on hospital cancer screening, admissions, interventions, length of stay, and mortality among prostate and colorectal cancer patients. We identified the areas in cancer patient journey that were majorly impacted by COVID-19 and further demonstrated that an estimated 2–16% monthly capacity increase would be required to clear all unperformed cancer procedures in a timely fashion. These system disruptions have profound implications for cancer management and ongoing care. To minimize the ongoing impact of COVID-19 on cancer services and disease progression, a multipronged approach involving all stakeholders to 1. clear the backlogs of treatment procedures and screening events and 2. reorganize our cancer services to create a resilient system to future crises and disruptions is urgently needed.

On average, a 4–8-week suspension of all non-urgent medical procedures was implemented across regions in March 2020 in Canada. A further 4-week suspension order was implemented in MB, ON, NL in November 2020, April 2021, and February 2021, respectively. These waves of postponement and cancellation of all non-urgent medical procedures including cancer screening and diagnostic interventions directly contributed to delayed cancer detection in the future. Additionally, cancer patients have been shown to be more vulnerable to worse clinical outcomes when infected with COVID-19 (Liang et al., 2020; Wu & McGoogan, 2020), further boosting patient’s reluctance to seek healthcare services out of fears of contracting COVID-19. Decreased physician referrals, shortages of staff and proper protective equipment during COVID-19 have also been shown to be a major contributing factor to decreased hospital admissions and cancer treatments (Mahase, 2020; Mayor, 2020; Wu et al., 2020).

Interestingly, a higher demand for imaging-based interventions, radiotherapies, and pharmacotherapies was observed in our datasets, suggesting a shift in cancer treatment paradigm during COVID-19. These alternative treatment options have gained patient preference perhaps to delay surgeries while the healthcare system is overwhelmed with COVID-19 cases. One national study showed that 46% of oncologists indicated they have modified treatment plans for more than 25% of their cancer patients (Yong et al., 2020). However, the clinical implications of these adaptations are poorly understood. Future systematic studies are warranted to investigate the magnitude of the risks associated with changes in cancer management including disruptions of usual care, delivering suboptimal care, and disruptions of clinical research etc. to minimize potential harms to cancer patients in a pandemic.

A recent simulation study has demonstrated that a 6-month screening interruption could lead to 2220 additional advanced colorectal cancer cases and an additional 960 cancer deaths in Canada (Yong et al., 2020). Although the majority of the missed cancer diagnoses was observed in the first wave of the pandemic, as of February 2022, oncologists across the nation are still reporting more advanced than usual cancer cases that normally would have been diagnosed sooner (Eskander et al., 2022; Schrag et al., 2020; Walker et al., 2021). More importantly, pandemic-related delays in cancer surgeries have been shown to be associated with 0.01 to 0.07 life-years lost per patient among cancer patients (Parmar et al., 2022). There is no doubt that the backlogs of delayed surgeries and cancer cases will take a significant toll on our already strained healthcare system in the years to come (Hanna et al., 2020a). In fact, the situation has been exacerbated by the loss of healthcare staff due to clinical burnout and high mental distress among primary care providers (Ting et al., 2022; Zador et al., 2022). In the short-term, embracing digital health developments such as telemedicine, remote monitoring, and virtual support programs for cancer patients have been proven effective in minimizing system level delays in cancer treatment as the pandemic evolves (Qian et al., 2020; Turco et al., 2022). Establishing designated diagnostic and treatment hubs will also be necessary for the unmet demand for cancer diagnosis and interventions. In the long-term, updating treatment guidelines for both physicians and patients will be critical to minimize health disparities at the age, gender, race, and regional level in cancer care as more advanced cancer cases show up in the coming years (Levit et al., 2020; Ratnapradipa et al., 2022).

The current study certainly has several limitations. First, our datasets only contain hospital data at the aggregate level and did not include data from private clinics and practices. We were also not able to obtain hospital data from BC and QC. However, recent studies have indicated that BC and QC experienced similar levels of system disruptions in cancer management (Agnihotram et al., 2022; Malagón et al., 2022). As a result, our results are likely conservative and represent an underestimation of the real numbers of screening events and hospital admissions. Second, our data grouping was limited by the half-year (April–September and October–March) data reporting structure, weekly or monthly data were not available. Additionally, hospital LOS was calculated for the entire visit of the patient for all diagnoses recorded during a hospitalization, therefore, LOS may not be associated with just one diagnosis. Nevertheless, our sample only included patients whose Most Responsible Diagnosis was either prostate or colorectal cancer. Lastly, it is important to acknowledge that the findings presented in this study is primarily observational in nature. As well, the waves of COVID-19 refer to significant surges of COVID-19 cases across Canada overall, however, differences in the timing and sizes may vary across different regions. Taken together, we believe that the current study will serve as an important baseline for future investigations aiming to examine the long-term effects of COVID-19 on cancer outcomes and extend these initial findings among prostate and colorectal cancer patients in Canada.

## Supplementary Information

Below is the link to the electronic supplementary material.

Supplementary file1 (DOCX 35 kb)

Supplementary file2 (XLSX 24 kb)

Supplementary file3 (DOCX 26 kb)

Supplementary file4 (DOCX 28 kb)

Supplementary file5 (DOCX 27 kb)

Supplementary file6 (DOCX 31 kb)

Supplementary file7 (DOCX 31 kb)

Supplementary file8 (DOCX 27 kb)

Supplementary file9 (DOCX 27 kb)

Supplementary file10 (DOCX 26 kb)
