# Supplementary material for: Impact of COVID-19 on hospital screening, diagnosis and treatment activities among prostate and colorectal cancer patients in Canada
Source: Int J Health Econ Manag. 2023 Apr 2;23(3):345–60. doi: 10.1007/s10754-023-09342-3 (PMC10067511; doi:10.1007/s10754-023-09342-3)
Supplement: Supplementary file 1 — Supplementary file1 (DOCX 35 kb) [file 10754_2023_9342_MOESM1_ESM.docx]

**Method**

**Results**

**Supplemental Table 1**

**Comparison1_CIHI.xlsx**

P Value Normality | Pre Data: 0.503 (Normal)

**Comparison2_CIHI.xlsx**

P Value Normality | Pre Data: 0.477 (Normal)

**Comparison3_CIHI.xlsx**

P Value Normality | Pre Data: 0.482 (Normal)

**Comparison4_CIHI.xlsx**

P Value Normality | Pre Data: 0.557 (Normal)

**Comparison5_CIHI.xlsx**

P Value Normality | Pre Data: 0.066 (Normal)

**Comparison6_CIHI.xlsx**

P Value Normality | Pre Data: 0.895 (Normal)

**Comparison7_CIHI.xlsx**

P Value Normality | Pre Data: 0.536 (Normal)

**Comparison8_CIHI.xlsx**

P Value Normality | Pre Data: 0.103 (Normal)

**Comparison9_CIHI.xlsx**

P Value Normality | Pre Data: 0.008 (Non-Normal)

**Comparison10_CIHI.xlsx**

P Value Normality | Pre Data: 0.202 (Normal)

**Comparison11_CIHI.xlsx**

P Value Normality | Pre Data: 0.378 (Normal)

**Comparison12_CIHI.xlsx**

P Value Normality | Pre Data: 0.303 (Normal)

**Comparison13_CIHI.xlsx**

P Value Normality | Pre Data: 0.051 (Normal)

**Comparison14_CIHI.xlsx**

P Value Normality | Pre Data: 0.917 (Normal)

**Supplemental Table 2**

**Comparison15_CIHI.xlsx**

P Value Normality | Pre Data: 0.380 (Normal)

**Comparison16_CIHI.xlsx**

P Value Normality | Pre Data: 0.872 (Normal)

**Comparison17_CIHI.xlsx**

P Value Normality | Pre Data: 0.372 (Normal)

**Comparison18_CIHI.xlsx**

P Value Normality | Pre Data: 0.220 (Normal)

**Comparison19_CIHI.xlsx**

P Value Normality | Pre Data: 0.581 (Normal)

**Comparison20_CIHI.xlsx**

P Value Normality | Pre Data: 0.142 (Normal)

**Comparison21_CIHI.xlsx**

P Value Normality | Pre Data: 0.562 (Normal)

**Comparison22_CIHI.xlsx**

P Value Normality | Pre Data: 0.132 (Normal)

**Comparison23_CIHI.xlsx**

P Value Normality | Pre Data: 0.983 (Normal)

**Comparison24_CIHI.xlsx**

P Value Normality | Pre Data: 0.135 (Normal)

**Comparison25_CIHI.xlsx**

P Value Normality | Pre Data: 0.586 (Normal)

**Comparison26_CIHI.xlsx**

P Value Normality | Pre Data: 0.361 (Normal)

**Comparison27_CIHI.xlsx**

P Value Normality | Pre Data: 0.000 (Non-Normal)

**Comparison28_CIHI.xlsx**

P Value Normality | Pre Data: 0.855 (Normal)

**Comparison29_CIHI.xlsx**

P Value Normality | Pre Data: 0.708 (Normal)

**Comparison30_CIHI.xlsx**

P Value Normality | Pre Data: 0.747 (Normal)

**Comparison31_CIHI.xlsx**

P Value Normality | Pre Data: 0.739 (Normal)

**Comparison32_CIHI.xlsx**

P Value Normality | Pre Data: 0.423 (Normal)

**Comparison33_CIHI.xlsx**

P Value Normality | Pre Data: 0.035 (Non-Normal)

**Supplemental Table 3**

**Comparison34_CIHI.xlsx**

P Value Normality | Pre Data: 0.784 (Normal)

**Comparison35_CIHI.xlsx**

P Value Normality | Pre Data: 0.155 (Normal)

**Comparison36_CIHI.xlsx**

P Value Normality | Pre Data: 0.999 (Normal)

**Comparison37_CIHI.xlsx**

P Value Normality | Pre Data: 0.409 (Normal)

**Comparison38_CIHI.xlsx**

P Value Normality | Pre Data: 0.918 (Normal)

**Comparison39_CIHI.xlsx**

P Value Normality | Pre Data: 0.478 (Normal)

**Comparison40_CIHI.xlsx**

P Value Normality | Pre Data: 0.110 (Normal)

**Comparison41_CIHI.xlsx**

P Value Normality | Pre Data: 0.332 (Normal)

**Comparison42_CIHI.xlsx**

P Value Normality | Pre Data: 0.002 (Non-Normal)

**Comparison43_CIHI.xlsx**

P Value Normality | Pre Data: 0.433 (Normal)

**Comparison44_CIHI.xlsx**

P Value Normality | Pre Data: 0.534 (Normal)

**Comparison45_CIHI.xlsx**

P Value Normality | Pre Data: 0.008 (Non-Normal)

**Comparison46_CIHI.xlsx**

P Value Normality | Pre Data: 0.189 (Normal)

**Comparison47_CIHI.xlsx**

P Value Normality | Pre Data: 0.619 (Normal)

**Comparison48_CIHI.xlsx**

P Value Normality | Pre Data: 0.417 (Normal)

**Comparison49_CIHI.xlsx**

P Value Normality | Pre Data: 0.040 (Non-Normal)

**Supplemental Table 4**

**Comparison50_CIHI.xlsx**

P Value Normality | Pre Data: 0.115 (Normal)

**Comparison51_CIHI.xlsx**

P Value Normality | Pre Data: 0.759 (Normal)

**Comparison52_CIHI.xlsx**

P Value Normality | Pre Data: 0.041 (Non-Normal)

**Comparison53_CIHI.xlsx**

P Value Normality | Pre Data: 0.638 (Normal)

**Comparison54_CIHI.xlsx**

P Value Normality | Pre Data: 0.204 (Normal)

**Comparison55_CIHI.xlsx**

P Value Normality | Pre Data: 0.412 (Normal)

**Comparison56_CIHI.xlsx**

P Value Normality | Pre Data: 0.327 (Normal)

**Comparison57_CIHI.xlsx**

P Value Normality | Pre Data: 0.000 (Non-Normal)

**Comparison58_CIHI.xlsx**

P Value Normality | Pre Data: 0.072 (Normal)

**Comparison59_CIHI.xlsx**

P Value Normality | Pre Data: 0.180 (Normal)

**Comparison60_CIHI.xlsx**

P Value Normality | Pre Data: 0.032 (Non-normal)

**Comparison61_CIHI.xlsx**

P Value Normality | Pre Data: 0.881 (Normal)

**Comparison62_CIHI.xlsx**

P Value Normality | Pre Data: 0.228 (Normal)

**Comparison63_CIHI.xlsx**

P Value Normality | Pre Data: 0.538 (Normal)

**Comparison64_CIHI.xlsx**

P Value Normality | Pre Data: 0.555 (Normal)

**Comparison65_CIHI.xlsx**

P Value Normality | Pre Data: 0.316 (Normal)

**Comparison66_CIHI.xlsx**

P Value Normality | Pre Data: 0.384 (Normal)

**Comparison67_CIHI.xlsx**

P Value Normality | Pre Data: 0.592 (Normal)

**Comparison68_CIHI.xlsx**

P Value Normality | Pre Data: 0.006 (Non-Normal)

**Comparison69_CIHI.xlsx**

P Value Normality | Pre Data: 0.101 (Normal)

**Comparison70_CIHI.xlsx**

P Value Normality | Pre Data: 0.050 (Normal)

**Comparison71_CIHI.xlsx**

P Value Normality | Pre Data: 0.013 (Non-Normal)

**Comparison72_CIHI.xlsx**

P Value Normality | Pre Data: 0.962 (Normal)

**Comparison73_CIHI.xlsx**

P Value Normality | Pre Data: 0.166 (Normal)

**Comparison74_CIHI.xlsx**

P Value Normality | Pre Data: 0.656 (Normal)

**Comparison75_CIHI.xlsx**

P Value Normality | Pre Data: 0.987 (Normal)

**Comparison76_CIHI.xlsx**

P Value Normality | Pre Data: 0.056 (Normal)

**Supplemental Table 5**

**Comparison77_CIHI.xlsx**

P Value Normality | Pre Data: 0.910 (Normal)

**Comparison78_CIHI.xlsx**

P Value Normality | Pre Data: 0.480 (Normal)

**Comparison79_CIHI.xlsx**

P Value Normality | Pre Data: 0.733 (Normal)

**Comparison80_CIHI.xlsx**

P Value Normality | Pre Data: 0.396 (Normal)

**Comparison81_CIHI.xlsx**

P Value Normality | Pre Data: 0.005 (Non-Normal)

**Comparison82_CIHI.xlsx**

P Value Normality | Pre Data: 0.005 (Non-Normal)

**Comparison83_CIHI.xlsx**

P Value Normality | Pre Data: 0.480 (Normal)

**Comparison84_CIHI.xlsx**

P Value Normality | Pre Data: 0.861 (Normal)

**Comparison85_CIHI.xlsx**

P Value Normality | Pre Data: 0.651 (Normal)

**Comparison86_CIHI.xlsx**

P Value Normality | Pre Data: 0.233 (Normal)

**Comparison87_CIHI.xlsx**

P Value Normality | Pre Data: 0.426 (Normal)

**Comparison88_CIHI.xlsx**

P Value Normality | Pre Data: 0.042 (Non-Normal)

**Comparison89_CIHI.xlsx**

P Value Normality | Pre Data: 0.221 (Normal)

**Comparison90_CIHI.xlsx**

P Value Normality | Pre Data: 0.051 (Normal)

**Comparison91_CIHI.xlsx**

P Value Normality | Pre Data: 0.317 (Normal)

**Comparison92_CIHI.xlsx**

P Value Normality | Pre Data: 0.030 (Non-Normal)

**Comparison93_CIHI.xlsx**

P Value Normality | Pre Data: 0.939 (Normal)

**Comparison94_CIHI.xlsx**

P Value Normality | Pre Data: 0.595 (Normal)

**Comparison95_CIHI.xlsx**

P Value Normality | Pre Data: 0.020 (Non-Normal)

**Comparison96_CIHI.xlsx**

P Value Normality | Pre Data: 0.246 (Normal)

**Comparison97_CIHI.xlsx**

P Value Normality | Pre Data: 0.519 (Normal)

**Supplemental Table 6**

**Comparison98_CIHI.xlsx**

P Value Normality | Pre Data: 0.367 (Non-Normal)

**Comparison99_CIHI.xlsx**

P Value Normality | Pre Data: 0.176 (Normal)

**Comparison100_CIHI.xlsx**

P Value Normality | Pre Data: 0.062 (Normal)

**Comparison101_CIHI.xlsx**

P Value Normality | Pre Data: 0.004 (Non-Normal)

**Comparison102_CIHI.xlsx**

P Value Normality | Pre Data: 0.505 (Normal)

**Comparison103_CIHI.xlsx**

P Value Normality | Pre Data: 0.404 (Normal)

**Comparison104_CIHI.xlsx**

P Value Normality | Pre Data: 0.763 (Normal)

**Comparison105_CIHI.xlsx**

P Value Normality | Pre Data: 0.000 (Non-Normal)

**Comparison106_CIHI.xlsx**

P Value Normality | Pre Data: 0.142 (Normal)

**Comparison107_CIHI.xlsx**

P Value Normality | Pre Data: 0.125 (Normal)

**Comparison108_CIHI.xlsx**

P Value Normality | Pre Data: 0.165 (Normal)

**Comparison109_CIHI.xlsx**

P Value Normality | Pre Data: 0.415 (Normal)

**Comparison110_CIHI.xlsx**

P Value Normality | Pre Data: 0.036 (Non-Normal)

**Comparison111_CIHI.xlsx**

P Value Normality | Pre Data: 0.001 (Non-Normal)

**Comparison112_CIHI.xlsx**

P Value Normality | Pre Data: 0.302 (Normal)

**Comparison113_CIHI.xlsx**

P Value Normality | Pre Data: 0.101 (Normal)

**Supplemental Table 7**

**Comparison114_CIHI.xlsx**

P Value Normality | Pre Data: 0.765 (Normal)

**Comparison115_CIHI.xlsx**

P Value Normality | Pre Data: 0.392 (Normal)

**Comparison116_CIHI.xlsx**

P Value Normality | Pre Data: 0.763 (Normal)

**Comparison117_CIHI.xlsx**

P Value Normality | Pre Data: 0.894 (Normal)

**Comparison118_CIHI.xlsx**

P Value Normality | Pre Data: 0.895 (Normal)

**Comparison119_CIHI.xlsx**

P Value Normality | Pre Data: 0.968 (Normal)

**Comparison120_CIHI.xlsx**

P Value Normality | Pre Data: 0.372 (Normal)

**Comparison121_CIHI.xlsx**

P Value Normality | Pre Data: 0.052 (Normal)

**Comparison122_CIHI.xlsx**

P Value Normality | Pre Data: 0.936 (Normal)

**Comparison123_CIHI.xlsx**

P Value Normality | Pre Data: 0.541 (Normal)

**Comparison124_CIHI.xlsx**

P Value Normality | Pre Data: 0.267 (Normal)

**Comparison125_CIHI.xlsx**

P Value Normality | Pre Data: 0.452 (Normal)

**Comparison126_CIHI.xlsx**

P Value Normality | Pre Data: 0.059 (Normal)

**Comparison127_CIHI.xlsx**

P Value Normality | Pre Data: 0.782 (Normal)

**Comparison128_CIHI.xlsx**

P Value Normality | Pre Data: 0.882 (Normal)

**Comparison129_CIHI.xlsx**

P Value Normality | Pre Data: 0.232 (Normal)

**Supplemental Table 8**

**Comparison130_CIHI.xlsx**

P Value Normality | Pre Data: 0.419 (Normal)

**Comparison131_CIHI.xlsx**

P Value Normality | Pre Data: 0.128 (Normal)

**Comparison132_CIHI.xlsx**

P Value Normality | Pre Data: 0.018 (Non-Normal)

**Comparison133_CIHI.xlsx**

P Value Normality | Pre Data: 0.069 (Normal)

**Comparison134_CIHI.xlsx**

P Value Normality | Pre Data: 0.000 (Non-Normal)

**Comparison135_CIHI.xlsx**

P Value Normality | Pre Data: 0.050 (Normal)

**Comparison136_CIHI.xlsx**

P Value Normality | Pre Data: 0.716 (Normal)

**Comparison137_CIHI.xlsx**

P Value Normality | Pre Data: 0.593 (Normal)

**Comparison138_CIHI.xlsx**

P Value Normality | Pre Data: 0.639 (Normal)

**Comparison139_CIHI.xlsx**

P Value Normality | Pre Data: 0.961 (Normal)

**Comparison140_CIHI.xlsx**

P Value Normality | Pre Data: 0.510 (Normal)

**Comparison141_CIHI.xlsx**

P Value Normality | Pre Data: 0.000 (Non-Normal)

**Comparison142_CIHI.xlsx**

P Value Normality | Pre Data: 0.063 (Normal)

**Comparison143_CIHI.xlsx**

P Value Normality | Pre Data: 0.956 (Normal)

**Comparison144_CIHI.xlsx**

P Value Normality | Pre Data: 0.003 (Non-Normal)

>
