# Supplementary material for: Impact of COVID-19 on hospital screening, diagnosis and treatment activities among prostate and colorectal cancer patients in Canada
Source: Int J Health Econ Manag. 2023 Apr 2;23(3):345–60. doi: 10.1007/s10754-023-09342-3 (PMC10067511; doi:10.1007/s10754-023-09342-3)
Supplement: Supplementary file 3 — Supplementary file3 (DOCX 26 kb) [file 10754_2023_9342_MOESM3_ESM.docx]

Supplemental Table 1. **Hospital Prostate and Colorectal Screening Events in AB/MB/SK, ON, and ATL between April 2017- March 2021.** Baseline data are presented as mean±SEM whereas first and second wave of COVID-19 data are presented as sum of the total screening events registered for the specified period. Asterisks indicate a statistically significant *p* value in a t test or Mann-Whitney U test comparison analysis where * = *p*<0.05, ** = *p*<0.01 and *** = *p*<0.0001. AB, Alberta; MB, Manitoba; SK, Saskatchewan; ON, Ontario; NS, Nova Scotia; PEI, Prince Edward Island; NB, New Brunswick; NL, Newfoundland and Labrador; N/R, None Reported.

| **Variable** | **# of Screening Events** | | | ***p*-value** (Baseline vs First wave of COVID-19) | ***p*-value** (Baseline vs Second wave of COVID-19) |
| --- | --- | --- | --- | --- | --- |
|  | Baseline  (April 2017-March 2020) | First wave of COVID-19  (April 2020-Sept 2020) | Second wave of COVID-19  (Oct 2020-March 2021) |  |  |
| **Prostate Cancer** | | | | | |
| **Region (province)** | | | | | |
| All regions | **146±22** | **74** | **98** | *p*=0.02* | *p*=0.08 |
| Prairies (AB/MB/SK) | 63±10 | 8 | 26 | *p*=0.003** | *p*=0.01* |
| ON | 53±9 | 41 | 47 | *p*=0.24 | *p*=0.53 |
| ATL (NS/PEI/NB/NL) | 31±7 | 25 | 25 | *p*=0.45 | *p*=0.45 |
|  |  |  |  |  |  |
| **Age (category), year** |  |  |  |  |  |
| <40 | N/R | N/R | N/R | - | - |
| 40-59 | 37±6 | 19 | 21 | *p*=0.04* | *p*=0.05 |
| 60-79 | 103±15 | 55 | 77 | *p*=0.02* | *p*=0.14 |
| 80+ | 7±3 | 0 | 0 | *p*=0.06 | *p*=0.06 |
|  |  |  |  |  |  |
| **Colorectal Cancer Cohort** | | | | | |
| **Region (province)** | | | | | |
| All regions | **67,345±2,047** | **25,835** | **50,314** | *p<*0.0001*** | *p*=0.004** |
| Prairies (AB/MB/SK) | 15,495±562 | 7,558 | 12,983 | *p<*0.0001*** | *p*=0.006** |
| ON | 47,692±1,393 | 16,438 | 33,086 | *p=*0.211 | *p=*0.211 |
| ATL (NS/PEI/NB/NL) | 4,159±175 | 1,839 | 4,245 | *p<*0.0001*** | *p*=0.64 |
|  |  |  |  |  |  |
| **Age (category), year** | | | | | |
| <40 | 2,692±39 | 1,449 | 2,604 | *p<*0.0001*** | *p*=0.07 |
| 40-59 | 30,864±1,179 | 10,688 | 21,475 | *p<*0.0001*** | *p<*0.0001*** |
| 60-79 | 32,504±954 | 13,031 | 25241 | *p<*0.0001*** | *p<*0.0001*** |
| 80+ | 1,286±41 | 667 | 994 | *p<*0.0001*** | *p<*0.0001*** |
