# Supplementary material for: Impact of COVID-19 on hospital screening, diagnosis and treatment activities among prostate and colorectal cancer patients in Canada
Source: Int J Health Econ Manag. 2023 Apr 2;23(3):345–60. doi: 10.1007/s10754-023-09342-3 (PMC10067511; doi:10.1007/s10754-023-09342-3)
Supplement: Supplementary file 4 — Supplementary file4 (DOCX 28 kb) [file 10754_2023_9342_MOESM4_ESM.docx]

Supplemental Table 2. **Prostate Cancer Hospital Admissions in AB/MB/SK, ON, and ATL between April 2017- March 2021.** Baseline data are presented as mean±SEM whereas first and second wave of COVID-19 data are presented as sum of the total hospital admissions registered for the specified period. Asterisks indicate a statistically significant *p* value in a t test or Mann-Whitney U test comparison analysis where * = *p*<0.05, ** = *p*<0.01 and *** = *p*<0.0001. AB, Alberta; MB, Manitoba; SK, Saskatchewan; ON, Ontario; NS, Nova Scotia; PEI, Prince Edward Island; NB, New Brunswick; NL, Newfoundland and Labrador; N/R, None Reported.

| **Variable** | **# of Hospital Admissions** | | | ***p*-value** (Baseline vs First wave of COVID-19) | ***p*-value** (Baseline vs Second wave of COVID-19) |
| --- | --- | --- | --- | --- | --- |
|  | Baseline  (April 2017-March 2020) | First wave of COVID-19  (April 2020-Sept 2020) | Second wave of COVID-19  (Oct 2020-March 2021) |  |  |
| **Prostate Cancer** | | | | | |
| **Region (province)** | | | | | |
| All regions | **6,779±75** | **5,599** | **6,520** |  |  |
| *Metastatic* | 902±16 | 817 | 790 | *p=*0.003** | *p=*0.001** |
| *Non-Metastatic* | 5,877±67 | 4,782 | 5,730 | *p*<0.0001*** | *p=*0.08 |
| Prairies (AB/MB/SK) | **1,764±51** | **1,547** | **1,740** |  |  |
| *Metastatic* | 260±8 | 232 | 221 | *p=*0.02* | *p=*0.005** |
| *Non-Metastatic* | 1,504±53 | 1,315 | 1,519 | *p=*0.02* | *p=*0.78 |
| ON | **4,003±61** | **3,212** | **3,708** |  |  |
| *Metastatic* | 572±21 | 517 | 503 | *p=*0.04* | *p=*0.02* |
| *Non-Metastatic* | 3,431±47 | 2,695 | 3,205 | *p*<0.0001*** | *p=*0.005** |
| ATL (NS/PEI/NB/NL) | **1,012±16** | **840** | **1,072** |  |  |
| *Metastatic* | 71±4 | 68 | 66 | *p=*0.55 | *p=*0.34 |
| *Non-Metastatic* | 941±17 | 772 | 1,006 | *p=*0.0002** | *p=*0.01* |
|  |  |  |  |  |  |
| **Age (category), year** |  |  |  |  |  |
| <40 | **1±0.9** | **0** | **0** |  |  |
| *Metastatic* | N/R | N/R | N/R | - | - |
| *Non-Metastatic* | 1±0.9 | 0 | 0 | *p=*1.00 | *p=*1.00 |
| 40-59 | **1,049±17** | **818** | **909** |  |  |
| *Metastatic* | 89±3 | 59 | 59 | *p=*0.0004** | *p=*0.0004** |
| *Non-Metastatic* | 960±17 | 759 | 850 | *p*<0.0001*** | *p=*0.001** |
| 60-79 | **4,683±66** | **3,906** | **4,619** |  |  |
| *Metastatic* | 506±18 | 478 | 434 | *p=*0.18 | *p=*0.01* |
| *Non-Metastatic* | 4,177±55 | 3,428 | 4,185 | *p*<0.0001*** | *p=*0.88 |
| 80+ | **1,047±8** | **875** | **992** |  |  |
| *Metastatic* | 308±6 | 280 | 297 | *p=*0.007** | *p=*0.15 |
| *Non-Metastatic* | 739±9 | 595 | 695 | *p=*0.207 | *p=*0.207 |
