# Supplementary material for: Impact of COVID-19 on hospital screening, diagnosis and treatment activities among prostate and colorectal cancer patients in Canada
Source: Int J Health Econ Manag. 2023 Apr 2;23(3):345–60. doi: 10.1007/s10754-023-09342-3 (PMC10067511; doi:10.1007/s10754-023-09342-3)
Supplement: Supplementary file 5 — Supplementary file5 (DOCX 27 kb) [file 10754_2023_9342_MOESM5_ESM.docx]

Supplemental Table 3. **Colorectal Cancer Hospital Admissions in AB/MB/SK, ON, and ATL between April 2017- March 2021.** Baseline data are presented as mean±SEM whereas first and second wave of COVID-19 data are presented as sum of the total hospital admissions registered for the specified period. Asterisks indicate a statistically significant *p* value in a t test or Mann-Whitney U test comparison analysis where * = *p*<0.05, ** = *p*<0.01 and *** = *p*<0.0001. AB, Alberta; MB, Manitoba; SK, Saskatchewan; ON, Ontario; NS, Nova Scotia; PEI, Prince Edward Island; NB, New Brunswick; NL, Newfoundland and Labrador.

| **Variable** | **# of Hospital Admissions** | | | ***p*-value** (Baseline vs First wave of COVID-19) | ***p*-value** (Baseline vs Second wave of COVID-19) |
| --- | --- | --- | --- | --- | --- |
|  | Baseline  (April 2017-March 2020) | First wave of COVID-19  (April 2020-Sept 2020) | Second wave of COVID-19  (Oct 2020-March 2021) |  |  |
| **Colorectal Cancer Cohort** | | | | | |
| **Region (province)** | | | | | |
| All regions | **12,226±82** | **10,057** | **11,901** |  |  |
| *Metastatic* | 3,389±56 | 2,963 | 3,082 | *p=*0.0003** | *p=*0.003** |
| *Non-Metastatic* | 15,616±96 | 12,693 | 15,339 | *p<*0.0001*** | *p=*0.03* |
| Prairies (AB/MB/SK) | **3,289±75** | **2,692** | **3,086** |  |  |
| *Metastatic* | 634±17 | 574 | 592 | *p=*0.02* | *p=*0.06 |
| *Non-Metastatic* | 2,655±74 | 2,118 | 2,476 | *p=*0.0008** | *p=*0.06 |
| ON | **7,437±47** | **6,270** | **7,417** |  |  |
| *Metastatic* | 1,563±39 | 1,324 | 1,400 | *p=*0.002** | *p=*0.008** |
| *Non-Metastatic* | 5,873±56 | 4,946 | 6,017 | *p<*0.0001*** | *p=*0.05 |
| ATL (NS/PEI/NB/NL) | **1,500±28** | **1,095** | **1,416** |  |  |
| *Metastatic* | 289±7 | 248 | 300 | *p=*0.002** | *p=*0.2 |
| *Non-Metastatic* | 1,210±28 | 847 | 1,116 | *p<*0.0001*** | *p=*0.02* |
|  |  |  |  |  |  |
| **Age (category), year** |  |  |  |  |  |
| <40 | **310±10** | **296** | **349** |  |  |
| *Metastatic* | 87±5 | 70 | 65 | *p=*0.168 | *p=*0.168 |
| *Non-Metastatic* | 223±7 | 226 | 284 | *p=*0.7 | *p=*0.0004** |
| 40-59 | **2,871±34** | **2,292** | **2,627** |  |  |
| *Metastatic* | 673±28 | 550 | 561 | *p=*0.007** | *p=*0.01* |
| *Non-Metastatic* | 2,197±10 | 1,742 | 2,066 | *p=*0.211 | *p=*0.211 |
| 60-79 | **6,494±71** | **5,294** | **6,504** |  |  |
| *Metastatic* | 1,261±24 | 1,093 | 1,222 | *p=*0.0009** | *p=*0.17 |
| *Non-Metastatic* | 5,233±86 | 4,201 | 5,282 | *p<*0.0001*** | *p=*0.59 |
| 80+ | **2,551±43** | **2,175** | **2,421** |  |  |
| *Metastatic* | 466±12 | 433 | 444 | *p=*0.04* | *p=*0.12 |
| *Non-Metastatic* | 2,085±34 | 1,742 | 1,977 | *p=*0.211 | *p=*0.211 |
