# Supplementary material for: Impact of COVID-19 on hospital screening, diagnosis and treatment activities among prostate and colorectal cancer patients in Canada
Source: Int J Health Econ Manag. 2023 Apr 2;23(3):345–60. doi: 10.1007/s10754-023-09342-3 (PMC10067511; doi:10.1007/s10754-023-09342-3)
Supplement: Supplementary file 6 — Supplementary file6 (DOCX 31 kb) [file 10754_2023_9342_MOESM6_ESM.docx]

Supplemental Table 4. **Prostate Cancer Treatment Activities in AB/MB/SK, ON, and ATL between April 2017- March 2021.** Baseline data are presented as mean±SEM whereas first and second wave of COVID-19 data are presented as sum of the total treatment events registered for the specified period. Asterisks indicate a statistically significant *p* value in a t test or Mann-Whitney U test comparison analysis where * = *p*<0.05, ** = *p*<0.01 and *** = *p*<0.0001. AB, Alberta; MB, Manitoba; SK, Saskatchewan; ON, Ontario; NS, Nova Scotia; PEI, Prince Edward Island; NB, New Brunswick; NL, Newfoundland and Labrador; N/R, None Reported.

| **Variable** | **# of Intervention Events** | | | ***p*-value** (Baseline vs First wave of COVID-19) | ***p*-value** (Baseline vs Second wave of COVID-19) |
| --- | --- | --- | --- | --- | --- |
|  | Baseline  (April 2017-March 2020) | First wave of COVID-19  (April 2020-Sept 2020) | Second wave of COVID-19  (Oct 2020-March 2021) |  |  |
| **Prostate Cancer Treatment Activities** | | | | | |
| **Region (province)** | | | | | |
| All regions | **6,164±83** | **5,243** | **6,464** |  |  |
| *Imaging (CT, Ultra, MRI)* | 1,487±33 | 1,315 | 1,675 | *p=*0.003** | *p=*0.002** |
| *Surgical Interventions* | 4,205±41 | 3,447 | 4,162 | *p<*0.0001*** | *p=*0.34 |
| *Radiotherapy* | 397±21 | 377 | 532 | *p=*0.453 | *p=*0.211 |
| *Pharmacotherapy* | 74±5 | 104 | 95 | *p=*0.002** | *p=*0.008** |
| Prairies (AB/MB/SK) | **1,229±45** | **1,170** | **1,319** |  |  |
| *Imaging (CT, Ultra, MRI)* | 183±20 | 194 | 232 | *p=*0.6 | *p=*0.06 |
| *Surgical Interventions* | 1,003±27 | 954 | 1,052 | *p=*0.13 | *p=*0.13 |
| *Radiotherapy* | 42±4 | 22 | 35 | *p=*0.003** | *p=*0.11 |
| *Pharmacotherapy* | 0.8±0.8 | 0 | 0 | *p=*1.00 | *p=*1.00 |
| ON | **3,453±65** | **2,844** | **3,518** |  |  |
| *Imaging (CT, Ultra, MRI)* | 723±29 | 601 | 758 | *p=*0.009** | *p=*0.29 |
| *Surgical Interventions* | 2,431±33 | 1,875 | 2,300 | *p<*0.0001*** | *p=*0.01* |
| *Radiotherapy* | 225±21 | 264 | 365 | *p=*0.453 | *p=*0.211 |
| *Pharmacotherapy* | 74±5 | 104 | 95 | *p=*0.001** | *p=*0.006** |
| ATL (NS/PEI/NB/NL) | **1,481±38** | **1,229** | **1,627** |  |  |
| *Imaging (CT, Ultra, MRI)* | 581±20 | 520 | 685 | *p=*0.03* | *p=*0.004** |
| *Surgical Interventions* | 771±13 | 618 | 810 | *p<*0.0001*** | *p=*0.03* |
| *Radiotherapy* | 130±7 | 91 | 132 | *p=*0.02* | *p=*0.73 |
| *Pharmacotherapy* | N/R | N/R | N/R | - | - |
|  |  |  |  |  |  |
| **Age (category), year** |  |  |  |  |  |
| <40 | **N/R** | **N/R** | **N/R** |  |  |
| *Imaging (CT, Ultra, MRI)* | N/R | N/R | N/R | - | - |
| *Surgical Interventions* | N/R | N/R | N/R | - | - |
| *Radiotherapy* | N/R | N/R | N/R | - | - |
| *Pharmacotherapy* | N/R | N/R | N/R | - | - |
| 40-59 | **1,089±22** | **883** | **991** |  |  |
| *Imaging (CT, Ultra, MRI)* | 228±8 | 194 | 229 | *p=*0.007** | *p=*0.92 |
| *Surgical Interventions* | 810±16 | 637 | 709 | *p=*0.0001** | *p=*0.0014** |
| *Radiotherapy* | 47±4 | 52 | 48 | *p=*0.19 | *p=*0.72 |
| *Pharmacotherapy* | 4±3 | 0 | 5 | *p=*0.755 | *p=*0.783 |
| 60-79 | **4,589±63** | **3,963** | **4,903** |  |  |
| *Imaging (CT, Ultra, MRI)* | 1,169±26 | 1,056 | 1,316 | *p=*0.006** | *p=*0.002** |
| *Surgical Interventions* | 3,043±35 | 2,552 | 3,094 | *p<*0.0001*** | *p=*0.2 |
| *Radiotherapy* | 341±17 | 294 | 467 | *p=*0.453 | *p=*0.211 |
| *Pharmacotherapy* | 36±3 | 61 | 26 | *p=*0.0005** | *p=*0.03* |
| 80+ | **486±9** | **397** | **570** |  |  |
| *Imaging (CT, Ultra, MRI)* | 90±5 | 65 | 130 | *p=*0.004** | *p=*0.0004** |
| *Surgical Interventions* | 352±7 | 258 | 359 | *p<*0.0001*** | *p=*0.37 |
| *Radiotherapy* | 10±3 | 31 | 17 | *p=*0.0009** | *p=*0.06 |
| *Pharmacotherapy* | 34±5 | 43 | 64 | *p=*0.12 | *p=*0.001** |
