# Supplementary material for: Impact of COVID-19 on hospital screening, diagnosis and treatment activities among prostate and colorectal cancer patients in Canada
Source: Int J Health Econ Manag. 2023 Apr 2;23(3):345–60. doi: 10.1007/s10754-023-09342-3 (PMC10067511; doi:10.1007/s10754-023-09342-3)
Supplement: Supplementary file 7 — Supplementary file7 (DOCX 31 kb) [file 10754_2023_9342_MOESM7_ESM.docx]

Supplemental Table 5. **Colorectal Cancer Treatment Activities in AB/MB/SK, ON, and ATL between April 2017- March 2021.** Baseline data are presented as mean±SEM whereas first and second wave of COVID-19 data are presented as sum of the total treatment events registered for the specified period. Asterisks indicate a statistically significant *p* value in a t test or Mann-Whitney U test comparison analysis where * = *p*<0.05, ** = *p*<0.01 and *** = *p*<0.0001. AB, Alberta; MB, Manitoba; SK, Saskatchewan; ON, Ontario; NS, Nova Scotia; PEI, Prince Edward Island; NB, New Brunswick; NL, Newfoundland and Labrador; N/R, None Reported.

| **Variable** | **# of Intervention Events** | | | ***p*-value** (Baseline vs First wave of COVID-19) | ***p*-value** (Baseline vs Second wave of COVID-19) |
| --- | --- | --- | --- | --- | --- |
|  | Baseline  (April 2017-March 2020) | First wave of COVID-19  (April 2020-Sept 2020) | Second wave of COVID-19  (Oct 2020-March 2021) |  |  |
| **Colorectal Cancer Cohort Treatment Activities** | | | | | |
| **Region (province)** | | | | | |
| All regions | **8,046±40** | **6,595** | **8,010** |  |  |
| *Imaging (CT, Xray)* | N/R | N/R | N/R | - | - |
| *Surgical Interventions* | 7,937±36 | 6,511 | 7,835 | *p<*0.0001*** | *p=*0.04* |
| *Radiotherapy* | 26±2 | 10 | 73 | *p=*0.001** | *p=*0.0001** |
| *Pharmacotherapy* | 83±15 | 74 | 102 | *p=*0.59 | *p=*0.25 |
| Prairies (AB/MB/SK) | **1,990±30** | **1,642** | **2,028** |  |  |
| *Imaging (CT, Xray)* | N/R | N/R | N/R | - | - |
| *Surgical Interventions* | 1,988±30 | 1,642 | 2,015 | *p<*0.0001*** | *p=*0.41 |
| *Radiotherapy* | 0±0 | 0 | 8 | - | - |
| *Pharmacotherapy* | 2±1 | 0 | 5 | *p=*0.755 | *p=*0.783 |
| ON | **4,851±67** | **4,103** | **4,833** |  |  |
| *Imaging (CT, Xray)* | N/R | N/R | N/R | - | - |
| *Surgical Interventions* | 4,749±65 | 4,019 | 4,676 | *p=*0.211 | *p=*1.00 |
| *Radiotherapy* | 26±2 | 10 | 65 | *p=*0.001** | *p<*0.0001*** |
| *Pharmacotherapy* | 76±13 | 74 | 92 | *p=*0.91 | *p=*0.27 |
| ATL (NS/PEI/NB/NL) | **1,205±24** | **850** | **1,149** |  |  |
| *Imaging (CT, Xray)* | N/R | N/R | N/R | - | - |
| *Surgical Interventions* | 1,200±24 | 850 | 1,144 | *p<*0.0001*** | *p=*0.06 |
| *Radiotherapy* | N/R | N/R | N/R | - | - |
| *Pharmacotherapy* | 5±2 | 0 | 5 | *p=*0.03* | *p=*0.92 |
|  |  |  |  |  |  |
| **Age (category), year** |  |  |  |  |  |
| <40 | **165±9** | **151** | **187** |  |  |
| *Imaging (CT, Xray)* | N/R | N/R | N/R | - | - |
| *Surgical Interventions* | 162±8 | 151 | 182 | *p=*0.24 | *p=*0.07 |
| *Radiotherapy* | N/R | N/R | N/R | - | - |
| *Pharmacotherapy* | 3±1 | 0 | 5 | *p=*0.581 | *p=*1.00 |
| 40-59 | **1,822±26** | **1,474** | **1,669** |  |  |
| *Imaging (CT, Xray)* | N/R | N/R | N/R | - | - |
| *Surgical Interventions* | 1,795±28 | 1,446 | 1,650 | *p<*0.0001*** | *p=*0.0035** |
| *Radiotherapy* | 5±2 | 0 | 5 | *p=*0.1 | *p=*0.89 |
| *Pharmacotherapy* | 22±6 | 28 | 14 | *p=*0.33 | *p=*0.27 |
| 60-79 | **4,543±47** | **3,678** | **4,649** |  |  |
| *Imaging (CT, Xray)* | N/R | N/R | N/R | - | - |
| *Surgical Interventions* | 4,482±41 | 3,622 | 4,542 | *p*=0.211 | *p=*1.00 |
| *Radiotherapy* | 11±3 | 10 | 24 | *p=*0.69 | *p=*0.01* |
| *Pharmacotherapy* | 50±9 | 46 | 83 | *p=*0.71 | *p=*0.01* |
| 80+ | **1,516±17** | **1,292** | **1,505** |  |  |
| *Imaging (CT, Xray)* | N/R | N/R | N/R | - | - |
| *Surgical Interventions* | 1,497±14 | 1,292 | 1,461 | *p=*0.211 | *p=*0.211 |
| *Radiotherapy* | 10±3 | 0 | 44 | *p=*0.03* | *p=*0.0002** |
| *Pharmacotherapy* | 9±2 | 0 | 0 | *p=*0.008** | *p=*0.008** |
