# Supplementary material for: Impact of COVID-19 on hospital screening, diagnosis and treatment activities among prostate and colorectal cancer patients in Canada
Source: Int J Health Econ Manag. 2023 Apr 2;23(3):345–60. doi: 10.1007/s10754-023-09342-3 (PMC10067511; doi:10.1007/s10754-023-09342-3)
Supplement: Supplementary file 8 — Supplementary file8 (DOCX 27 kb) [file 10754_2023_9342_MOESM8_ESM.docx]

Supplemental Table 6. **Median Calculated Length of Stay for Prostate Cancer Patients in AB/MB/SK, ON, and ATL between April 2017- March 2021.** Data are presented as mean±SEM. Asterisks indicate a statistically significant *p* value in a t test or Mann-Whitney U test analysis where * = *p*<0.05, ** = *p*<0.01 and *** = *p*<0.0001. AB, Alberta; MB, Manitoba; SK, Saskatchewan; ON, Ontario; NS, Nova Scotia; PEI, Prince Edward Island; NB, New Brunswick; NL, Newfoundland and Labrador.

| **Variable** | **Median Calculated LOS (days)** | | | ***p*-value** (Baseline vs First wave of COVID-19) | ***p*-value** (Baseline vs Second wave of COVID-19) |
| --- | --- | --- | --- | --- | --- |
|  | Baseline  (April 2017-March 2020) | First wave of COVID-19  (April 2020-Sept 2020) | Second wave of COVID-19  (Oct 2020-March 2021) |  |  |
| **Prostate Cancer** | | | | | |
| **Region (province)** | | | | | |
| All regions | **4.0±0.2** | **3.5** | **3.2** |  |  |
| *Metastatic* | 6.2±0.4 | 4.7 | 4.9 | *p=*0.211 | *p=*0.211 |
| *Non-Metastatic* | 1.9±0.2 | 2.2 | 1.5 | *p=*0.08 | *p=*0.06 |
| Prairies (AB/MB/SK) | **4.8±0.6** | **3.6** | **3.2** |  |  |
| *Metastatic* | 7.4±1.2 | 5.3 | 4.9 | *p=*0.15 | *p=*0.1 |
| *Non-Metastatic* | 2.2±0.5 | 2.0 | 1.5 | *p=*0.795 | *p=*0.611 |
| ON | **3.1±0.1** | **3.3** | **3.0** |  |  |
| *Metastatic* | 4.4±0.1 | 4.6 | 4.5 | *p=*0.09 | *p=*0.52 |
| *Non-Metastatic* | 1.8±0.2 | 2.0 | 1.5 | *p=*0.5 | *p=*0.21 |
| ATL (NS/PEI/NB/NL) | **4.3±0.3** | **3.6** | **3.4** |  |  |
| *Metastatic* | 6.9±0.6 | 4.3 | 5.4 | *p=*0.007** | *p=*0.05 |
| *Non-Metastatic* | 1.7±0.0 | 2.9 | 1.5 | *p*<0.0001*** | *p=*0.001** |
|  |  |  |  |  |  |
| **Age (category), year** |  |  |  |  |  |
| <40 | **2.3±0.8** | **0.7** | **0.2** |  |  |
| *Metastatic* | 3.3±1.5 | 0.0 | 0.3 | *p=*0.09 | *p=*0.12 |
| *Non-Metastatic* | 1.4±0.6 | 1.3 | 0.0 | *p=*0.87 | *p=*0.07 |
| 40-59 | **3.0±0.2** | **2.6** | **1.8** |  |  |
| *Metastatic* | 3.9±0.5 | 3.2 | 1.7 | *p=*0.18 | *p=*0.005** |
| *Non-Metastatic* | 2.1±0.1 | 2.0 | 2.0 | *p=*0.24 | *p=*0.24 |
| 60-79 | **4.1±0.1** | **3.7** | **3.6** |  |  |
| *Metastatic* | 6.1±0.2 | 5.3 | 5.2 | *p=*0.449 | *p=*0.449 |
| *Non-Metastatic* | 2.1±0.1 | 2.0 | 2.0 | *p=*0.752 | *p=*0.752 |
| 80+ | **6.8±0.3** | **7.1** | **7.3** |  |  |
| *Metastatic* | 11.2±0.7 | 10.3 | 12.5 | *p=*0.11 | *p=*0.28 |
| *Non-Metastatic* | 2.0±0.0 | 3.8 | 2.0 | *p*<0.0001*** | *p=*0.97 |
