# Supplementary material for: Impact of COVID-19 on hospital screening, diagnosis and treatment activities among prostate and colorectal cancer patients in Canada
Source: Int J Health Econ Manag. 2023 Apr 2;23(3):345–60. doi: 10.1007/s10754-023-09342-3 (PMC10067511; doi:10.1007/s10754-023-09342-3)
Supplement: Supplementary file 9 — Supplementary file9 (DOCX 27 kb) [file 10754_2023_9342_MOESM9_ESM.docx]

Supplemental Table 7. **Median Calculated Length of Stay for Colorectal Cancer Patients in AB/MB/SK, ON, and ATL between April 2017- March 2021.** Data are presented as mean±SEM. Asterisks indicate a statistically significant *p* value in a t test or Mann-Whitney U test comparison analysis where * = *p*<0.05, ** = *p*<0.01 and *** = *p*<0.0001. AB, Alberta; MB, Manitoba; SK, Saskatchewan; ON, Ontario; NS, Nova Scotia; PEI, Prince Edward Island; NB, New Brunswick; NL, Newfoundland and Labrador.

| **Variable** | **Median Calculated LOS (days)** | | | ***p*-value** (Baseline vs First wave of COVID-19) | ***p*-value** (Baseline vs Second wave of COVID-19) |
| --- | --- | --- | --- | --- | --- |
|  | Baseline  (April 2017-March 2020) | First wave of COVID-19  (April 2020-Sept 2020) | Second wave of COVID-19  (Oct 2020-March 2021) |  |  |
| **Colorectal Cancer Cohort** | | | | | |
| **Region (province)** | | | | | |
| All regions | **7.2±0.1** | **6.8** | **7.2** |  |  |
| *Metastatic* | 8.1±0.1 | 7.4 | 8.7 | *p=*0.007** | *p=*0.008** |
| *Non-Metastatic* | 6.2±0.1 | 6.1 | 5.8 | *p=*0.22 | *p=*0.007** |
| Prairies (AB/MB/SK) | **7.6±0.2** | **7.4** | **7.0** |  |  |
| *Metastatic* | 8.5±0.3 | 8.1 | 7.9 | *p=*0.27 | *p=*0.13 |
| *Non-Metastatic* | 6.7±0.2 | 6.8 | 6.1 | *p=*0.74 | *p=*0.03* |
| ON | **6.2±0.1** | **6.1** | **6.9** |  |  |
| *Metastatic* | 7.1±0.2 | 7.3 | 8.8 | *p=*0.6 | *p=*0.0004* |
| *Non-Metastatic* | 5.2±0.1 | 5.0 | 5.0 | *p=*0.14 | *p=*0.14 |
| ATL (NS/PEI/NB/NL) | **7.7±0.2** | **6.7** | **7.9** |  |  |
| *Metastatic* | 8.6±0.4 | 7.0 | 9.5 | *p=*0.009** | *p=*0.06 |
| *Non-Metastatic* | 6.7±0.1 | 6.5 | 6.2 | *p=*0.14 | *p=*0.02* |
|  |  |  |  |  |  |
| **Age (category), year** |  |  |  |  |  |
| <40 | **5.8±0.3** | **5.1** | **8.0** |  |  |
| *Metastatic* | 7.0±0.7 | 5.2 | 11.0 | *p=*0.05 | *p=*0.002** |
| *Non-Metastatic* | 4.7±0.2 | 5.0 | 5.0 | *p=*0.12 | *p=*0.12 |
| 40-59 | **6.4±0.1** | **6.0** | **5.6** |  |  |
| *Metastatic* | 7.4±0.3 | 6.6 | 6.5 | *p=*0.04* | *p=*0.03* |
| *Non-Metastatic* | 5.4±0.1 | 5.4 | 4.8 | *p=*0.33 | *p<*0.0001*** |
| 60-79 | **7.1±0.1** | **6.6** | **6.9** |  |  |
| *Metastatic* | 7.9±0.2 | 7.4 | 7.9 | *p=*0.04* | *p=*0.98 |
| *Non-Metastatic* | 6.2±0.1 | 5.9 | 5.9 | *p=*0.02* | *p=*0.02* |
| 80+ | **9.3±0.3** | **9.3** | **8.5** |  |  |
| *Metastatic* | 10.1±0.3 | 10.5 | 9.6 | *p=*0.33 | *p=*0.23 |
| *Non-Metastatic* | 8.5±0.3 | 8.0 | 7.3 | *p=*0.16 | *p=*0.02* |
