# Supplementary material for: Impact of COVID-19 on hospital screening, diagnosis and treatment activities among prostate and colorectal cancer patients in Canada
Source: Int J Health Econ Manag. 2023 Apr 2;23(3):345–60. doi: 10.1007/s10754-023-09342-3 (PMC10067511; doi:10.1007/s10754-023-09342-3)
Supplement: Supplementary file 10 — Supplementary file10 (DOCX 26 kb) [file 10754_2023_9342_MOESM10_ESM.docx]

Supplemental Table 8. **Hospital Prostate and Colorectal Mortality in AB/MB/SK, ON, and ATL between April 2017- March 2021.** Baseline data are presented as mean±SEM whereas first and second wave of COVID-19 data are presented as sum of the total patient expiration events for the specified period. Asterisks indicate a statistically significant *p* value in a t test or Mann-Whitney U test comparison analysis where * = *p*<0.05, ** = *p*<0.01 and *** = *p*<0.0001. AB, Alberta; MB, Manitoba; SK, Saskatchewan; ON, Ontario; NS, Nova Scotia; PEI, Prince Edward Island; NB, New Brunswick; NL, Newfoundland and Labrador; N/R, None Reported.

| **Variable** | **# of Patient Expirations** | | | ***p*-value** (Baseline vs First wave of COVID-19) | ***p*-value** (Baseline vs Second wave of COVID-19) |
| --- | --- | --- | --- | --- | --- |
|  | Baseline  (April 2017-March 2020) | First wave of COVID-19  (April 2020-Sept 2020) | Second wave of COVID-19  (Oct 2020-March 2021) |  |  |
| **Prostate Cancer Mortality** | | | | | |
| **Region (province)** | | | | | |
| All regions | **83±4** | **71** | **77** | *p=*0.03* | *p=*0.19 |
| Prairies (AB/MB/SK) | 30±3 | 22 | 23 | *p=*0.04* | *p=*0.06 |
| ON | 42±1 | 39 | 38 | *p=*0.295 | *p=*0.195 |
| ATL (NS/PEI/NB/NL) | 11±2 | 10 | 16 | *p=*0.69 | *p=*0.05 |
|  |  |  |  |  |  |
| **Age (category), year** |  |  |  |  |  |
| <40 | N/R | N/R | N/R | - | - |
| 40-59 | 1±1 | 0 | 0 | *p=*1.00 | *p=*1.00 |
| 60-79 | 37±2 | 35 | 34 | *p=*0.3 | *p=*0.16 |
| 80+ | 45±4 | 36 | 43 | *p=*0.1 | *p=*0.69 |
|  |  |  |  |  |  |
| **Colorectal Cancer Mortality** | | | | | |
| **Region (province)** | | | | | |
| All regions | **266±6** | **228** | **213** | *p=*0.002** | *p=*0.0004** |
| Prairies (AB/MB/SK) | 72±3 | 63 | 52 | *p=*0.04* | *p=*0.002** |
| ON | 160±6 | 151 | 131 | *p=*0.16 | *p=*0.004** |
| ATL (NS/PEI/NB/NL) | 33±2 | 14 | 30 | *p=*0.0002** | *p=*0.14 |
|  |  |  |  |  |  |
| **Age (category), year** | | | | | |
| <40 | 1±1 | 0 | 0 | *p=*1.00 | *p=*1.00 |
| 40-59 | 28±4 | 26 | 19 | *p=*0.63 | *p=*0.08 |
| 60-79 | 127±4 | 99 | 98 | *p=*0.0006** | *p=*0.0005** |
| 80+ | 111±3 | 103 | 96 | *p=*0.445 | *p=*0.445 |
